# Contrasting responses of root morphology and root-exuded organic acids to low phosphorus availability in three important food crops with divergent root traits

**DOI:** 10.1093/aobpla/plv097

**Published:** 2015-08-17

**Authors:** Yan-Liang Wang, Marit Almvik, Nicholas Clarke, Susanne Eich-Greatorex, Anne Falk Øgaard, Tore Krogstad, Hans Lambers, Jihong Liu Clarke

**Affiliations:** 1 Norwegian Institute of Bioeconomy Research (NIBIO), PO Box 115, N-1431 Ås, Norway; 2 Department of Environmental Sciences, Norwegian University of Life Sciences, PO Box 5003, N-1432 Ås, Norway; 3 School of Plant Biology and Institute of Agriculture, University of Western Australia, Crawley (Perth), WA 6009, Australia

**Keywords:** Crops, hydroponic culture, phosphorus, root exudates, root morphology

## Abstract

Phosphorus (P) is an important element for crop productivity and is widely applied in fertilizers. Most P fertilizers applied to land are sorbed onto soil particles, so research on improving plant uptake of less easily available P is important. In the current study, we investigated the responses in root morphology and root-exuded organic acids (OAs) to low available P (1 μM P) and sufficient P (50 μM P) in barley, canola and micropropagated seedlings of potato—three important food crops with divergent root traits, using a hydroponic plant growth system. We hypothesized that the dicots canola and tuber-producing potato and the monocot barley would respond differently under various P availabilities. WinRHIZO and liquid chromatography triple quadrupole mass spectrometry results suggested that under low P availability, canola developed longer roots and exhibited the fastest root exudation rate for citric acid. Barley showed a reduction in root length and root surface area and an increase in root-exuded malic acid under low-P conditions. Potato exuded relatively small amounts of OAs under low P, while there was a marked increase in root tips. Based on the results, we conclude that different crops show divergent morphological and physiological responses to low P availability, having evolved specific traits of root morphology and root exudation that enhance their P-uptake capacity under low-P conditions. These results could underpin future efforts to improve P uptake of the three crops that are of importance for future sustainable crop production.

## Abstract (precis)

Available phosphorus (P) is one of the most important factors affecting crop production worldwide. Study on improving plant P uptake is hence of global importance. We have investigated the responses of root morphology and root-exuded organic acids to low P availability in three important food crops (barley, canola and potato) with divergent root traits using a hydroponic culture system. Results showed that plants evolved divergent adaptations of root morphology and exudation as a response to low P availability. These results could underpin future efforts to improve P uptake of the three crops which are important for future sustainable crop production.

## Introduction

Phosphorus (P) deficiency is one of the major limitations for crop productivity globally. In modern agriculture, mineral P fertilizers are extensively used, and large economic benefits have been generated. However, P fertilizers have also been added in amounts that far exceed the amount of P removed at harvest: up to ∼90 % of P applied as fertilizer may be strongly sorbed to the soil to become less available to plants, or eroded and lost in run-off (Gerke *et al*. 1994; Smith and Schindler 2009; Andersson *et al*. 2013). As a consequence, high P input has produced many problems, such as eutrophication and hypoxia of waterways (Withers *et al*. 2001) as well as eutrophication of oligotrophic natural terrestrial systems (Lambers *et al*. 2013). Hence, it is important to understand P-acquisition mechanisms and genetically improve crop plants to be able to acquire P more efficiently, which would enable reduced P-fertilizer application.

Higher plants have developed various responses to low P availability, including modified gene expression, morphological responses especially in root architecture (e.g. reduced primary root length, more lateral roots and root hairs) and physiological modification of the rhizosphere by root exudation and pH changes, as well as metabolic responses (Raghothama 1999; Lynch 2011). To improve P-acquisition efficiency, understanding changes in root structure and activities under various P availabilities is necessary. During the past decades, three main types of changes have been revealed: (i) changes in root morphology, such as root length, root hairs, root distribution and root diameter (Williamson *et al*. 2001; López-Bucio *et al*. 2002; Lambers *et al*. 2006; Desnos 2008); (ii) modification of root physiology, important for release of water and protons into soil and exudation of nucleases, acid phosphatases and carboxylic acids to change soil properties and mobilize organic and inorganic P sources (Read *et al*. 2003; Lambers *et al*. 2006, 2008; Prieto *et al*. 2012) and (iii) colonization by arbuscular mycorrhizal fungi, since P uptake by the mycorrhizal hyphae is the dominant pathway for P acquisition when plant roots are colonized by arbuscular mycorrhizal fungi (Smith *et al*. 2003). Finally, these factors might not operate alone but interact with each other to improve P uptake.

Although significant progress has been made in understanding plant processes associated with soil P mobilization and acquisition, each plant species is different and likely responds to P supply differently. Furthermore, there are a number of issues that are not well understood, such as the complex coordination of root morphology, and physiological and biochemical responses under variable P availability (Shen *et al*. 2011). Root exudation represents a significant carbon cost to plants, and root exudates are influenced by plant age, species and genotype, root structure and environmental factors including both biotic and abiotic stressors (Badri and Vivanco 2009). Phosphorus deficiency increases the release of organic acids (OAs) by roots of certain plants (López-Bucio *et al*. 2000), which could play a key role in mobilizing P from mineral surfaces and from oxides and hydroxides of Al and Fe, as well as Ca-phosphates (Neumann and Römheld 2001; Ryan *et al*. 2001; Neumann *et al*. 2014).

In this study, we aimed to investigate contrasting responses of root morphology and root exudation with regards to acquiring P. Therefore, we selected three economically important crops with different root systems as plant materials: barley, canola and potato. Barley, a typical monocot, has a wide-ranging network of fibrous roots, whereas the dicots (canola and potato) tend to have a long tap-root with thick lateral roots. Barley and potato are capable of forming an arbuscular mycorrhizal symbiosis to acquire soil P, while the non-mycorrhizal canola cannot do this (Brundrett 2009; Lambers and Teste 2013). We compared the root structure and root-exuded OAs of these three crop plants under different P supply using hydroponic culture. We hypothesized that under low P, (1) canola has a more developed root system with longer roots, greater root surface area and more lateral roots, or increased root exudates to compensate for its lack of mycorrhizas; (2) barley and potato exude less OAs than canola and thus save carbon for releasing other compounds to promote rhizosphere microbial activity and (3) compared with canola and barley, potato releases the least OAs and stores most of its belowground carbon resources in tubers as starch. The information generated in this study will be of value for a more sustainable production of canola, barley and potato.

## Methods

### Plant material and experimental design

Canola (*Brassica napus* cv. MARIE), barley (*Hordeum vulgare* cv. HEDER) and micropropagated seedlings of potato (*Solanum tuberosum* cv. PIMPERNEL) were selected for this study. Hydroponic culture experiments were conducted in the greenhouse of the Norwegian University of Life Sciences (NMBU). Germinated canola and barley seeds were first grown in full-strength nutrient solution containing 1.5 mM KCl, 2 mM Ca(NO_3_)_2_, 1.0 mM MgSO_4_, 1 μM H_3_BO_3_, 1 μM MnSO_4_, 1 μM ZnSO_4_, 0.5 μM CuSO_4_, 0.37 μM Na_2_MoO_4_ and 50 μM Fe-EDTA. Phosphorus was added as KH_2_PO_4_ in a concentration of 1 or 50 μM, designated as low P availability (P1) and P sufficient (P50), respectively (concentrations used by Cheng *et al*. 2014). Five-day-old uniform seedlings without seed residues were then carefully transferred to 1-L plastic pots (one plant per pot) filled with nutrient solution containing P1 or P50, with pH adjusted to 5.8 ± 0.2. Solutions were replaced every third day; at the same time, pots were rearranged randomly. For potato, ∼10 cm heights of tissue culture-derived seedlings were selected for hydroponic culture, and no root tuber was produced in this 4-week experiment. Plants were grown at 25 °C/16 °C day/night temperature with a 16-h photoperiod at a light intensity of 200 ± 20 μmol m^−2^ s^−1^ and 50–75 % relative humidity. Three independent replications were carried out.

### Root exudate collection

Root exudates were collected as described by Khorassani *et al*. (2011). Briefly, after plants had been grown hydroponically under P1 or P50 for 2 and 4 weeks, whole root systems of intact plants were carefully washed with deionized water to remove the nutrient solution. Then the whole root system was placed into ultrapure Milli-Q water (Millipore, Billerica, MA, USA) in a container to collect root exudates; the volume varied from 20 to 50 mL depending on the size of the root system. The roots were kept in the water for 2 h (between 10:00 and 13:00) under the same controlled-climate conditions as described for plant growth. Neumann and Römheld (2001) reported that roots are not harmed by Milli-Q water and no significant degradation of the exudates occurs in a short time period of 2 h. As Valentinuzzi *et al*. (2015) reported, water is the most effective and suitable trap solution to collect exudates like OAs, especially in a short time period such as 2 h. Micropur (0.01 g L^−1^, Katadyn Products, Kemptthal, Switzerland) was then added to the solution to inhibit the activity of microorganisms (Cheng *et al*. 2014). The collected root exudates were immediately frozen at −20 °C. Before analysis with liquid chromatography triple quadrupole mass spectrometry (LC–MS/MS), collected root exudate samples were filtered with Phenex regenerated cellulose syringe filters (pore size: 0.45 µm, filter diameter: 15 mm) (Phenomenex, Torrance, CA, USA), and 0.2 µg deuterium-labelled succinic acid was added to each sample to be used as an internal standard (IS).

### Determination of OAs released from roots

The OA analysis was performed using a Waters Alliance 2695 LC system coupled to a Quattro Ultima Pt triple quadrupole mass spectrometer (Micromass, Manchester, UK), equipped with an electrospray ionization (ESI) source. The auto-sampler in the LC system cooled the samples to 5 °C. The injection volume was 50 µL and sample constituents were separated using an Acquity XSelect HSS T3 (50 × 3 mm, 2.5 µm) analytical column (Waters, Milford, MA, USA) at a column oven temperature of 40 °C and a sample run time of 6 min. The trifunctional alkyl C18-bonded phase used for the HSS T3 sorbent is compatible with the 100 % aqueous mobile phase; so, aqueous formic acid (0.08 M, pH 2.4) was used as the sole mobile phase solution at a flow rate of 0.3 mL min^−1^. Electrospray ionization conditions were as follows: capillary voltage of 3.0 kV, source temperature of 120 °C, desolvation temperature of 350 °C, cone gas flow of 80 L h^−1^ and desolvation gas flow of 620 L h^−1^. The cone voltage, collision energy and selection of product ions were optimized for each OA for maximum intensities. Two characteristic fragmentations of the OA precursor ion ([M−H]^−^) were monitored. The most abundant product ion was used for quantification, and the second product ion was used for verification of the OA (Table 1). The optimal quantifier ions in our MS system were identical to the quantifier ions used by Erro *et al*. (2009). For malonic acid, which has a small molecular mass, only one product ion was used. Pure OA standards (citric acid (>99 %), l-(−) malic acid (>99.5 %), succinic acid (>99.5 %), malonic acid (99 %), l-(+)-tartaric acid (>99.5 %) and deuterium-labelled succinic acid-2,2,3,3-d_4_ (98 %)) were purchased from Sigma-Aldrich (St Louis, MO, USA). The OAs were mixed in Milli-Q water in the range 0.05–2.0 µg mL^−1^, containing a fixed amount (µg mL^−1^) of the IS deuteriated succinic acid. Internal standard calibration was performed using weighted (1/*x*) quadratic regression analysis of the peak area ratios (analyte/IS) versus the concentration ratios. Limits of quantification were 0.015–0.04 μg mL^−1^, corresponding to signal-to-noise ratios of 15.

**Table 1.** Multiple reaction monitoring (MRM) conditions for the LC-ESI-MS/MS analysis of OAs.

OA	Retention time (min)	MRM transitions; precursor > product ions	Cone voltage (V)	Collision energy (V)
Tartaric acid	1.15	149 > 87 + 73	35	12
Malic acid	1.30	133 > 115 + 71	35	12
Malonic acid	1.45	103 > 59	35	6
Citric acid	1.91	191 > 111 + 87	35	18
Succinic acid	2.43	117 > 73 + 99	35	12
IS succinic acid-d_4_	2.39	121 > 77 + 102	35	12

### Plant harvest and measurements of root morphology

Plants were harvested 28 days after transfer to hydroponics, and roots and shoots were sampled separately for subsequent analysis. The total number of green leaves and senesced leaves was recorded. Root length and root surface area were measured using WinRHIZO (EPSON 1680, WinRHIZO Pro2003b, Regent Instruments Inc., Quebec, Canada). Shoot and root dry weight (DW) were measured separately after being oven-dried for 48 h at 65 °C.

### Determination of P

Total P concentrations in dry root and shoot tissues were determined by inductively coupled plasma atomic emission spectroscopy (AtomComp 1100, Thermo Jarrell-Ash, MA, USA) according to Ogner *et al*. (1999) after digestion in a mixture of 65 % (v/v) HNO_3_/72 % (v/v) HClO_4_ (5 : 1, v/v) at 220 °C in a microwave oven.

### Statistical analyses

Data were statistically analysed by R software (version 3.1.3; software and commanders were downloaded from the NMBU library). Two-way ANOVAs were used to study main effects of P level, species and their interaction on all the parameters involved in this study. For multiple comparisons to determine which of the six P/species combinations were significantly different from each other, *post hoc* pair-wise Tukey honest significant difference tests were used after ANOVA. For all analyses, the significance *α* level of 0.05 was used.

## Results

### Plant growth at different P availability

**Figure 1.**
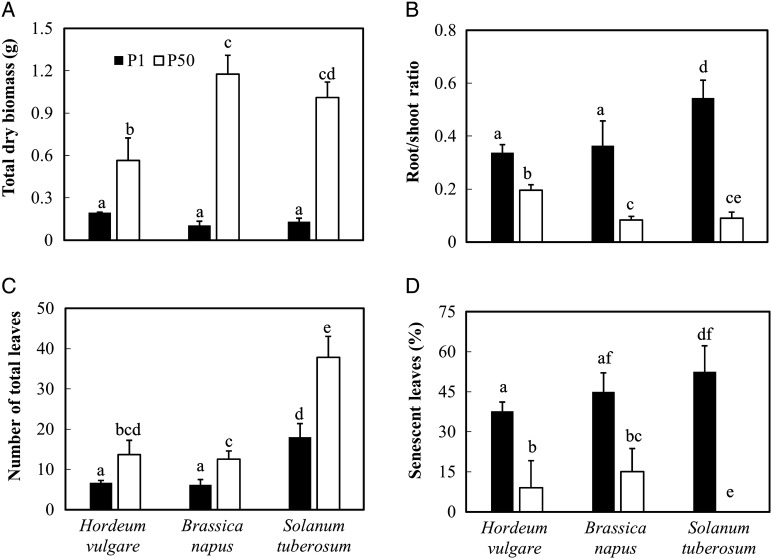
(A) Total dry biomass, (B) the root : shoot DW ratio, (C) total leaf number and (D) percentage senesced leaves of *H. vulgare*, *B. napus* and *S. tuberosum* grown in nutrient solution at two levels of P supply (P1 = 1 μM P, P50 = 50 μM P). Plants were harvested after 28 days growth. Values are means ± SE of *n* = 9. Different letters indicate significant differences (*P* < 0.05) as shown by Tukey tests.

After 4 weeks of P1 treatment, a 65 % lower total dry biomass was found in barley and >87 % lower biomass in canola and potato, compared with plants grown under P50; no significant differences were found between these three crops at a low P level, while canola and potato showed 102 and 68 % more biomass than barley at P50, respectively (Fig. 1A; Table 2). The root : shoot DW ratio increased considerably in plants grown under P1, with >70 % increase in barley, and >300 and 400 % increase in canola and potato, respectively; at P50, barley showed a significantly greater (∼100 %) root/shoot ratio than canola and potato (Fig. 1B; Table 2). Consistent with the results for biomass, the number of total leaves was decreased by ∼42–53 % at P1 compared with that at P50 for all three crops; potato (with an average of 38 leaves at P50 and 18 leaves at P1) had more leaves than canola (12 leaves at P50 and 7 leaves at P1) and barley (13 leaves at P50 and 7 leaves at P1) under both P50 and P1 conditions (Fig. 1C; Table 2). Low P availability also led to earlier leaf senescence in all three species, especially in potato (>53 % senescent leaves), with many shed leaves after 3 weeks, while there was almost no leaf senescence in potato at P50 (Fig. 1D; Table 2).

**Table 2.** *F* and *P* values of a two-way ANOVA on the effects of P level (P1 versus P50), species (barley, canola and potato) and their interactions on the parameters determined in this study. P, P level; S, species; df, degrees of freedom; Error, error df values; D15, day 15; D28, day 28.

Parameters	Factors	df	*F*	*P*
Total DW	P	1	1048.1	<0.001
	S	2	46.7	<0.001
	P × S	2	83.8	<0.001
	Error	48		
Root/shoot ratio	P	1	397.2	<0.001
	S	2	11.0	<0.001
	P × S	2	27.4	<0.001
	Error	48		
Number of total leaves	P	1	245.4	<0.001
	S	2	248.2	<0.001
	P × S	2	36.9	<0.001
	Error	48		
Percentage of senescent leaves	P	1	546.2	<0.001
	S	2	6.1	0.004
	P × S	2	20.8	<0.001
	Error	48		
Total root length	P	1	2.2	0.154
	S	2	1.9	0.173
	P × S	2	34.9	<0.001
	Error	24		
Root surface area	P	1	32.0	<0.001
	S	2	3.6	0.045
	P × S	2	36.4	<0.001
	Error	24		
Root tip number	P	1	0.01	0.91
	S	2	11.8	<0.001
	P × S	2	22.9	<0.001
	Error	24		
D15 Citric acid	P	1	7.7	0.009
	S	2	6.3	0.017
	P × S	2	13.8	0.001
	Error	32		
D15 Malic acid	P	1	45.6	<0.001
	S	2	3.6	0.036
	P × S	2	1.7	0.199
	Error	48		
D15 Succinic acid	P	1	2.5	0.123
	S	2	9.6	<0.001
	P × S	2	8.1	0.001
	Error	48		
D28 Citric acid	P	1	47.3	<0.001
	S	2	28.4	<0.001
	P × S	2	23.7	<0.001
	Error	32		
D28 Malic acid	P	1	22.4	<0.001
	S	2	9.5	<0.001
	P × S	2	8.9	0.001
	Error	48		
D28 Succinic acid	P	1	7.7	0.008
	S	2	11.9	<0.001
	P × S	2	15.2	<0.001
	Error	48		
Shoot P concentration	P	1	1419.2	<0.001
	S	2	92.3	<0.001
	P × S	2	91.4	<0.001
	Error	30		
Shoot P content	P	1	676.1	<0.001
	S	2	1.1	0.340
	P × S	2	0.43	0.654
	Error	30		
Root P concentration	P	1	264.8	<0.001
	S	2	19.4	<0.001
	P × S	2	12.8	<0.001
	Error	30		
Root P content	P	1	127.5	<0.001
	S	2	8.4	0.001
	P × S	2	6.0	0.006
	Error	30

### Root morphology

**Figure 2.**
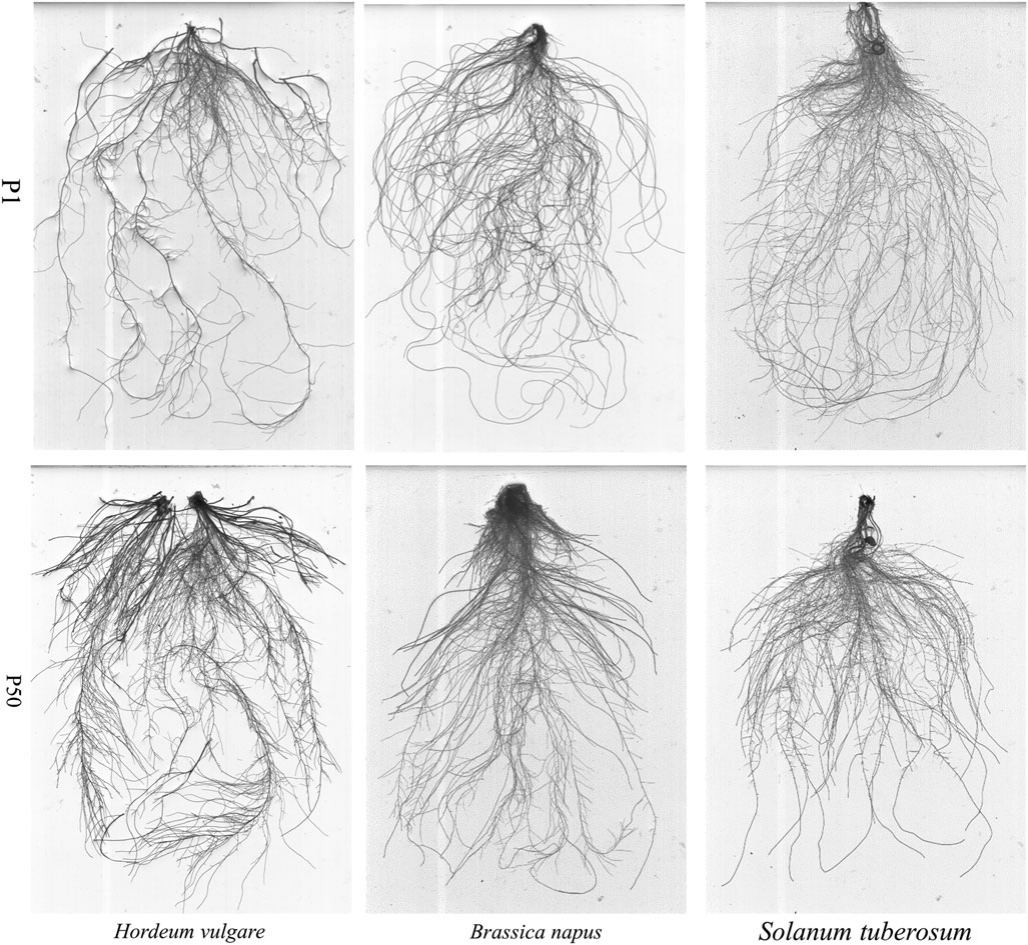
Root structure of *H. vulgare*, *B. napus* and *S. tuberosum* grown in nutrient solution at two levels of P supply (P1 = 1 μM P, P50 = 50 μM P). Plants were harvested after 28 days growth. Photos were taken by a WinRHIZO scanner and one representative photo of each crop was selected.

**Figure 3.**
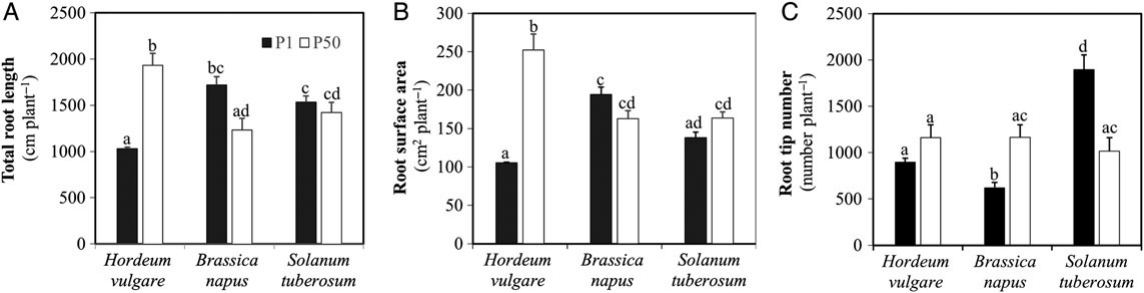
(A) Total root length, (B) root surface area and (C) root tip number of *H. vulgare*, *B. napus* and *S. tuberosum* grown in nutrient solution at two levels of P supply (P1 = 1 μM P, P50 = 50 μM P). Plants were harvested after 28 days growth. Values are means ± SE of *n* = 6. Different letters indicate significant differences (*P* < 0.05) as shown by Tukey tests.

Changes in root morphology varied significantly among the studied crop species (Figs 2 and 3; Table 2). Under P1, compared with P50, barley showed a marked reduction in total root length (44 %) and root surface area (58 %), whereas canola showed a significant increase in total root length (37 %), but a decrease in number of root tips (45 %). Total root length in potato and root surface area in canola did not change significantly. For potato, the number of root tips almost doubled under P deficiency. At P1, canola and potato showed 58 and 49 % greater total root length than barley, respectively; canola had 18 % greater root surface area than potato, and potato had 57 % greater root surface area than barley, but the average number of root tips decreased in the order potato (1892) > barley (883) > canola (627). On the other hand, barley showed 36 and 55 % greater root length than potato and canola, respectively, and ∼55 % greater root surface area than canola and potato at P50. These three crops had almost the same number of root tips at P50.

### Root exudate analysis

**Figure 4.**
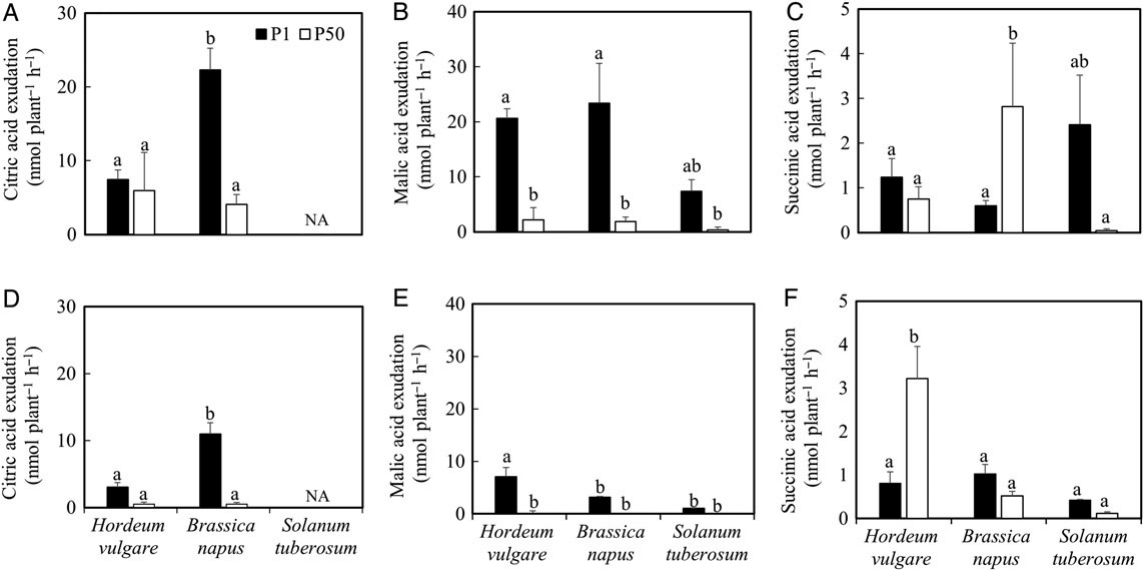
(A and D) Citric acid exudation, (B and E) malic acid exudation and (C and F) succinic acid exudation of *H. vulgare*, *B. napus* and *S. tuberosum* grown in nutrient solution at two levels of P supply (P1 = 1 μM P, P50 = 50 μM P). Root exudates were collected after 15 days growth (A–C) and 28 days growth (D–F). Values are means ± SE of *n* = 8–9. Different letters indicate significant differences (*P* < 0.05) as shown by Tukey tests. NA, not available.

Three OAs—citric, malic and succinic acids—were detected in the root exudates of both P1 and P50 plants (Fig. 4; Table 2). All these three OAs were found in canola and barley exudates, while citric acid was not detected in potato root exudates (Fig. 4A and D). Under P1 treatment, the greatest amounts of total OAs (citric + malic + succinic acids) were found in canola root exudates (54 nmol plant^−1^ h^−1^), while potato plants released the lowest amount of OAs (11 nmol plant^−1^ h^−1^). When root exudates were collected on the 15th day (just after solutions were replaced), P-deficient canola plants exuded over 400 % more citric acid and 1300 % more malic acid than the plants with sufficient P supply (Fig. 4A and B). However, succinic acid exudation at P1 in canola was only ∼25 % of that at P50 (Fig. 4C). The exudation of all three OAs increased in the P1 treatment of barley, but for citric and succinic acids, this increase was not significant (Fig. 4A–C). Canola roots exuded over three times more citric acid than barley roots under P deficiency (Fig. 4A). For potato, the amounts of released malic and succinic acid were in general only 10–50 % of those for barley or canola (except for succinic acid at P1), and no significant differences between P1 and P50 were found (Fig. 4B and C). When root exudates were collected on the 28th day (just before harvesting), the three studied OAs released by roots in all studied crops decreased dramatically, with ∼30–70 % reduction compared with those collected on the 15th day (Fig. 4D–F), and canola exuded the greatest amounts of citric acid, while barley exuded the greatest amounts of malic acid under P1 conditions; at P50, barley released significantly more succinic acid than canola and potato did (∼3.2 nmol plant^−1^ h^−1^ in barley versus 0.6 nmol plant^−1^ h^−1^ in canola and 0.1 nmol plant^−1^ h^−1^ in potato).

### Phosphorus status in shoots and roots

**Figure 5.**
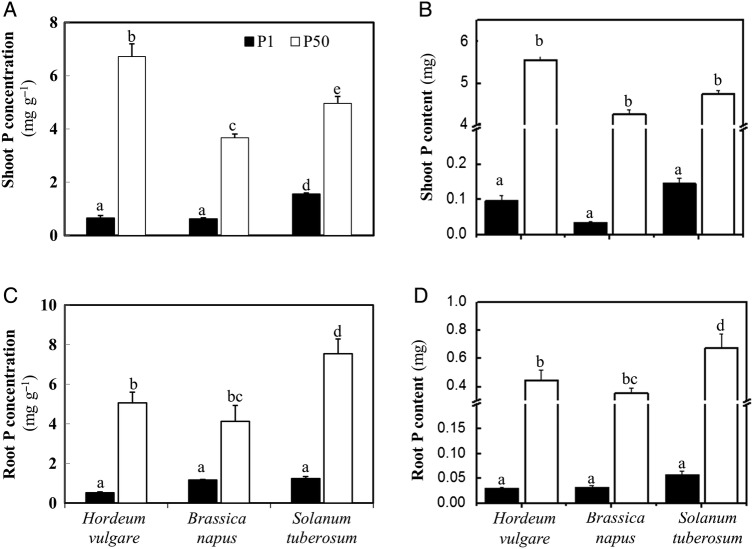
(A) Phosphorus concentration in shoot tissue, (B) P content in shoot tissue, (C) P concentration in root tissue and (D) P content in root tissue of *H. vulgare*, *B. napus* and *S. tuberosum* grown in nutrient solution at two levels of P supply (P1 = 1 μM P, P50 = 50 μM P). Plants were harvested after 28 days growth. Values are means ± SE of *n* = 6–7. Different letters indicate significant differences (*P* < 0.05) as shown by Tukey tests.

Unsurprisingly, the P concentrations and P contents in both shoot and root tissues were significantly higher (between 3 and 81 times) for plants supplied with P50 compared with those of plants supplied with P1 (Fig. 5; Table 2). Under P50 supply, the P concentrations and P contents in shoots decreased in the order barley > potato > canola (Fig. 5A and B), while in roots, the order was potato > barley > canola (Fig. 5C and D). The shoot and root P concentrations varied from 4 to 8 mg g^−1^ DW, comparing well with a P concentration in agricultural crops that generally varies from 1 to 5 mg P g^−1^ DW (Anonymous 1999). This shows that 50 μM was a P concentration sufficient for plant growth. Under P1 conditions, potato had the highest P concentrations and P contents in shoots, although no statistically significant difference was found in shoot P content. Canola had shoot P concentrations equal to those of barley, but it had a P content that was only 45 % as high due to having only 55 % as much shoot biomass (Figs 1A and 5A and B). In root tissues, potato and canola had almost the same P concentrations, which were 121 % higher (*P* < 0.05, Student's *t*-test) than in barley (Fig. 5C), and potato roots showed the greatest P content (0.06 mg versus 0.03 mg P in barley and canola), but these differences were not significant (Fig. 5D).

## Discussion

Phosphorus availability and uptake directly affect crop productivity. Understanding the relationships between root architecture, exudates and P availability was thus the main objective of this study. To understand the complex adaptive responses of root morphology and root exudates to low P availability, we carried out hydroponic culture experiments on canola, barley and potato, three crop species with different root systems. Although a hydroponic culture system does not reflect natural growth conditions for plants compared with a soil experiment, it is widely used (Gahoonia *et al*. 2000; Dechassa and Schenk 2004; Ligaba *et al*. 2004; Wang *et al*. 2013; Cheng *et al*. 2014) and useful for studying root architecture, including tiny root tips, and for collecting root exudates for biochemical analysis of OAs. Furthermore, the effects of plant–plant interactions were minimized in our system, since we grew a single plant per pot. Contrasting responses of root morphology and root-exuded OAs to low P availability in these three important food crops were revealed. These results could underpin future efforts to improve P uptake of the three crops.

### The role of root morphology in improving P uptake

Root architecture plays an important role in P uptake. Previous studies have shown that at reduced P availability, most species allocate more biomass to roots and allocate root biomass in shallow soil horizons, as well as increase root length and develop more and longer root hairs and lateral roots; some even produce cluster roots, thereby promoting P uptake (Nielsen *et al*. 2001; Lambers *et al*. 2006, 2015; Brown *et al*. 2013). In this study, our results showed a pronounced increase in root/shoot biomass ratio for the three studied species (Fig. 1B) under low P supply, which corresponds well with previous reports (Hermans *et al*. 2006; Hammond and White 2008). However, we found some differences in root traits like root length, lateral root numbers and root surface area among different crops (Fig. 3): under low P availability, barley showed a 44 % reduction in root length, 58 % smaller root surface area and 21 % lower root tip number; canola showed a 37 % increase in root length and a 45 % reduction in root tips and potato showed a doubling of the number of root tips, compared with under P50 conditions. However, Steingrobe *et al*. (2001) observed enhanced root-length production at a low P supply in barley in a field experiment, which differs from our results. This could be due to the two different methodologies used (i.e. we used a hydroponic culture, whereas Steingrobe *et al*. performed field experiments involving a more complicated environment around the roots). Moreover, the effects of micropropagated plantlets on root systems are not clear so far.

Phosphorus uptake by plants is dependent on the surface area and length of the root system and on lateral roots to explore a large soil volume (Richardson *et al*. 2009; Balemi and Negisho 2012; Lambers *et al*. 2015). In our study, as we used KH_2_PO_4_ as P source, root morphology plays a dominant role in P acquisition. At P50, barley had greater shoot P concentrations and P contents than canola and potato (Fig. 5A and B), probably due to its significantly longer total root length and greater root surface area, enabling greater P uptake (Fig. 3A and B), because these three crops had the same root tip number (Fig. 3C). Another explanation is its smaller biomass and higher root/shoot ratio (Fig. 1A and B), resulting in higher shoot P concentrations compared with those of canola and potato. On the other hand, potato showed a significantly greater root P concentration and P content than barley and canola did at P50 (Fig. 5C and D), suggesting that potato roots may have a greater P uptake capacity than barley and canola. Further studies are needed to reveal the mechanisms for this. In addition, under low P supply, canola roots showed greater root length and larger root surface area than barley and potato, while potato showed a remarkable increase in root tips compared with that at P50, and hence had greater root P concentrations and contents than canola and barley (Fig. 3). This also suggests that root tips had a major effect on improving P uptake, as found by Fitter *et al*. (2002). However, no significant differences were found in root P concentrations and P contents under P1, which could be due to the very limited P supply. Our results for potato root architecture agree with those of McArthur and Knowles (1993*a*), who reported that root growth in potato is less influenced by P deficiency than either leaf or stem growth. Furthermore, they found a greater root colonization level of arbuscular mycorrhizal fungi for P-stressed potato plants, and P uptake by roots was enhanced by different kinds of arbuscular mycorrhizal fungi at all levels of P supply (McArthur and Knowles 1993*b*). Brundrett (2002) reviewed the major influence that root morphology characteristics such as cortex cell properties and root features like root length/biomass ratio, root branching and root hairs have on mycorrhiza formation. Highly mycorrhiza-dependent plants tend to have coarser root systems and would not be as responsive to changes in nutrient availability. Hence, these root traits of potato, especially abundant root tips, might favour mycorrhizal colonization.

### The possible role of root exudation in low-P responses

Root exudation of OAs is considered as an important mechanism to mobilize P sources and alleviate P starvation, because OAs can replace organic and inorganic P that is bound to soil particles (Lambers *et al*. 2006). The fact that those plants that release more OAs could take up more P from growth media and soil has also been confirmed using genetically modified plants (Lü *et al*. 2012; Wang *et al*. 2013). However, root exudates are influenced by many factors (Dechassa and Schenk 2004). We used the most suitable and effective trap solution—water—to collect the exudates (Valentinuzzi *et al*. 2015), but we could not completely avoid some microbial breakdown, so the root-exuded OAs were possibly somewhat underestimated in our data (Kuijken *et al*. 2015).

Great differences in OA exudates were found among species in our system (Fig. 4). For canola, the absolute rate of citric acid exudation corresponded well with that reported by Hoffland *et al*. (1989). Furthermore, an increase in citric and malic acids and a decrease in succinic acid were found for canola under P starvation, which compares well with the results of Hoffland *et al*. (1989), except that malic acid was the dominant OA. On the other hand, no OAs were induced by P deficiency from canola roots, as reported by Ligaba *et al*. (2004) using the same system.

For barley, citric, acetic and fumaric acids were detected in root exudates of two cultivars under P deficiency by Gahoonia *et al*. (2000) using a hydroponic culture, but they only showed a difference between two cultivars; no information about the difference between P supply and P deficiency was given. Our results showed no significant difference in citric acid exudation in barley between P-sufficient and P-deficient plants, but there was an increase in malic acid exudation in the P deficiency treatment (Fig. 4A, B and E), thus showing that there are differences in the types and quantities of OAs that are released from different crop species and varieties in response to P starvation.

For potato, few references about OA exudates can be found in the literature, but the increase in succinic acid exudation in response to P deficiency was in accordance with the results of Dechassa and Schenk (2004), while the absolute value in our results was lower (∼0.002 versus 0.36 nmol (cm root)^−1^ h^−1^). The reason for this difference is unclear.

The decrease in the OA amounts released by roots of P-stressed plants after 4 weeks (Fig. 4D–F) suggests that root exudation was influenced by plant age (Dechassa and Schenk 2004; Badri and Vivanco 2009).

Citric acid has the strongest ability to release soil P (Ryan *et al*. 2001), malic acid is ∼10 times less effective than citric acid in mobilizing soil P and succinic acid complexes metal cations only very weakly and, hence, has only a relatively weak ability to release soil P (Nagarajah *et al*. 1970; Jones and Darrah 1995). Therefore, canola, with its relatively rapid exudation of citric acid, is likely to have the greatest ability among the investigated crops to mobilize insoluble soil P. These results provide good evidence to support our Hypothesis (1), but the effect of root exudates on P uptake could not be assessed in this study, because P was only added to the system as aqueous KH_2_PO_4_. Further investigation is required to reveal the effect of root exudates (OAs) on P mobilization and uptake from soil.

## Conclusions

Using a hydroponic culture system and various analytical methods, we have tested our hypotheses that canola, a dicot oilseed crop; potato, a tuber-producing dicot and barley, a monocot, respond differently under various levels of P availability. Our results revealed that the non-mycorrhizal species, canola, showed rapid rates of carboxylic acid exudation, which, together with its greater root length and root surface area, could allow canola to acquire poorly available P forms and explore a larger soil volume. The monocot and mycorrhizal species barley showed a reduction in root length and root surface area as well as low amounts of root exudates; in soil, this would result in a low P-uptake capacity. Potato released only trace amounts of root exudates but produced double the number of root tips under low-P conditions, which would benefit its P uptake in soil. Hence, our study indicates that plants evolved divergent adaptations of root morphology and exudation as a response to low P availability. The results and information generated in this study are valuable for future effective utilization of P and improving the productivity of the three important crops.

## Sources of Funding

This study was supported by the core funding of the strategic institute program on ‘Opportunities for sustainable use of phosphorus in food production’ at the Norwegian Institute of Bioeconomy Research.

## Contributions by the Authors

Y.-L.W., N.C. and J.L.C. designed the experiment. Y.-L.W. and M.A. conducted the experiment. All authors contributed to analysis of data, discussions and manuscript writing.

## Conflict of Interest Statement

None declared.
